# Prognostic factors in sudden hearing loss

**Published:** 2008-08-15

**Authors:** R Enache, I Sarafoleanu

**Affiliations:** ENT Department ‘Sfanta Maria’ Hospital, BucharestRomania

## Abstract

Sudden hearing loss is a sensorineural hypoacusis, unilateral in most of the cases, with an incidence peak in young adult. This article is the result of a four years retrospective study, in which we have tried to see how age, severity of hearing loss, presence or absence of vertigo, the timing of treatment initiation can influence the hearing recovery.

47 patients were included in this inpatient study. They were investigated (clinical, lab and imaging studies) and the treatment protocol included vasodilators, anti–inflammatory agents, vitamins, plasma expander.

In conclusion, a good prognosis in the hearing recovery was associated with absence of vertigo, early treatment (the first 7 days), and hearing loss less than 50dB. Age had no influence upon the recovery process.

## Introduction

Sudden hearing loss (SHL) is a sudden decrease or loss of hearing defined on audiometric criteria based on severity and time course. A commonly used criterion for diagnosis is a sensorineural hypoacusis of greater than 30dB over 3 contiguous audiometric frequencies. It was first described in the literature by De Klevn in 1944.

Female–to–male distribution appears to be almost equal. People of all age groups are affected, with a peak incidence between 30–60 years. Fortunately, the majority of cases of SHL are unilateral. Bilateral hearing loss occurs in approximately 1–2% of cases.

The epidemiology for sudden hearing loss has four theoretical pathways: a viral infection, a vascular compromise, an intracochlear membrane rupture (theory favored by Simmons in 1968 [1] and Goodhill in 1979 [2]) and immune–mediated inner ear disease (first described by McCabe in 1979 [3]).

The auditory system is complex and it depends on the normal function of the middle ear, cochlea and the central nervous system. Also, the hearing can be influenced by the metabolic, vascular, and endocrine systems. Any damage of one of these systems can affect the inner ear. So, sudden hearing loss can have many possible etiologies:

- infection (bacterial: meningitis, Scarlet fever; viral: mumps, varicella, cytomegalovirus);
- inflammation (sarcoidosis, Wegener granulomatosis, Cogan syndrome);
- vascular (hypercoagulable states, thrombosis or embolism of the internal audit artery, hypertension);
- tumor (acoustic neuroma, metastasis);
- trauma (temporal bone fracture, acoustic trauma);
- toxins (amino glycoside, cisplatin);
- endocrine abnormalities (hypothyroidism)

The symptoms appear sudden. Most patients report a hearing loss within minutes to several hours. The firs and most common symptom is deafness. It can be accompanied by tinnitus (80%), vertigo, headache, queasiness.

Even if the diagnosis of sudden hearing loss is easy at a first glance, in reality it is more complicated especially in those cases when other otic disorders coexist or when the patient can't say the symptom inception.

## Material and methods

A 4 years retrospective study through which we wanted to see how age, severity of hearing loss, presence or absence of vertigo, the timing of the initiation of treatment can influence the hearing recovery.

47 patients were included in this inpatient study. We underlined the characteristics related to patient's age, gender, associated pathology, the symptoms, the timing of treatment and the hearing recovery.

**Fig. 1:**
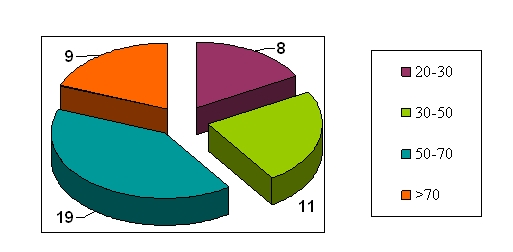
Age groups

People of all age groups were affected, with a peak between 50–70 years. (Fig. 1)

Gender repartition was: 49% females and 51% males.

**Fig. 2:**
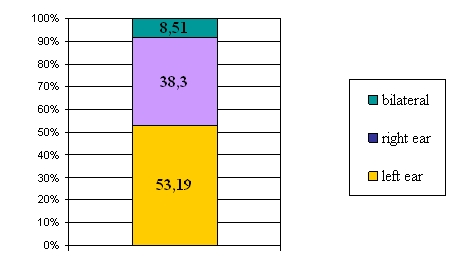
Hypoacusis type

Almost all patients presented as a first symptom sudden hypoacusis. Fortunately, the majority of cases were unilateral (left ear 53,19%, right ear 38,3%) (see Fig. 2). Patients also presented tinnitus, vertigo (8 cases), headache and queasiness.

It is said that there is a connection between vascular risk factors and the ones who can give a sudden deafness [4]. Hypercholesterolemia, smoking, cardiovascular pathology are risk factors for a sudden hearing loss. Pathologic anamnesis revealed that 28 patients had such a risk.

**Fig. 3:**
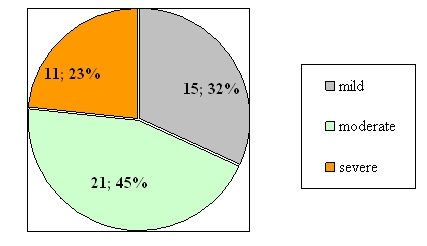
Hypoacusis severity

In the primary evaluation and the diagnosis each patient made a tonal audiogram, which we have repeated during and after the treatment, as a final evaluation, so we could evaluate the treatment efficiency and the hearing recovery. (see Fig. 3).

Depending on patients' symptoms and associated pathology, we have done some tests like: acoustic evoked potentials, echo Doppler and brain CT or MRI (in those cases in which the auditory tests were pathologic).

We used a ten days treatment with vasodilators (Pentoxifylline 200mg per day), anti–inflammatory agents (Cortisone 300–400mg per day), vitamins (B1, B6, and Calcium), and plasma expander 500mg per day. In 5 cases we also administrated antiviral agents and in 2 cases heparin. Eustachian tube permeabilisation maneuvers were used in 5 patients. Due to the economic reasons we could not use the hyperbaric oxygen therapy.

## Results and discussions

Patients' evolution under treatment was different. Complete recovery of hearing was found in 20 cases (42,55%). 18 patients (38,3%) had a 10–40dB recovery on the tonal audiogram but without reaching the normal standards, and 9 patients with severe hypoacusis had no recovery.

Analysis of recovery rates according to specific measurements of the severity of hearing loss, revealed a significant difference in favor of those with less severe deafness. Unster et al reported that prognosis for recovery was very poor in patients whose hearing loss was total. Shiraishi [5] also reported a complete, moderate, or slight recovery in 38% patients with a severe hearing loss. Our findings were consistent with those in the literature. No recovery in 9 patients with severe hypoacusis.

Our study revealed that a hearing loss of more than 50–60dB on audiogram, even with a good treatment, presented a moderate recovery no greater than 20–30dB. Greater the hearing loss, the worse is the recovery prognosis, because it reveals an irreversible lesion.

Hearing recovery is also influenced by the timing of the treatment initiation [5]. Earlier treatment (the first 7 days) was associated with a better prognosis, even a complete recovery.

The presence of vertigo was a negative prognosis factor. Moskowitz et al [6] reported that the presence of vertigo indicates a poor prognosis; they found that only 14% of patients they studied achieved a complete recovery of hearing. In our study, 8 patients were vertigo–positive, and none of them presented a good recovery.

Vertigo shows a damage of the vestibulocochlear pathways. For a good diagnosis an electronystagmography or an electrocochleography can be made. If we think that the mechanism could be a vascular one, an angio–MRI could be useful. The auditory evoked potentials, brain response time–locked to some ‘event’ in this case a sound, are also very important. Otoacoustic emissions tests can also be used in the diagnosis of SHL, their primary purpose being to determinate cochlear status.

The hearing recovery in patients with a cardiovascular pathology, a stroke in their antecedents or a hypertension peak, was modest.

Recovery was independent of age. 

The treatment regimens of SHL are varied and this diversity reflects both the different etiologies and the uncertainty in diagnosis. The protocol, which we also used in our department, included vasodilators, anti–inflammatory agents, vitamins, plasma expander, antiviral agents and heparin.

Theoretically, vasodilators improve the blood supply to the cochlea, reversing hypoxia. There are studies made on the real effect of Pentoxifylline and Ginkgo biloba, but none of them have provided its benefit versus placebo. Certain studies showed that βhistine reduces the cochlear hypoxia and improves the hearing [7].

The use of Dextran (plasma expander) has also been suggested as a means of creating a hypervolemic hemodilution, this causing a decrease in blood viscosity and an increase in cochlear blood flow [8].

Hypoxia goes to an inflammatory reaction with a release of histamine, eicosanoids, and prostaglandins. Corticosteroids are the primary anti–inflammatory agents used to treat SHL. The mechanism of action of cortisone is reduction of cochlear and auditory nerve inflammation [6,9]. In a recent study [10], intratympanic injection of Dexamethasone is shown to effectively improve hearing in patients with severe sudden hearing loss after treatment failure with standard therapy (Ho, 2005)[11].

After six days of treatment patients were evaluated with a tonal audiogram. If there was no change in their hearing features the treatment was stopped. Also, if there was a hearing recovery we administrated Ginkgo biloba and betahistine.

The treatment after the acute faze of the disease, at those patients with risks, consisted from Magnesium, recent studies showing that it may be good in preventing the SHL. Also, the role of antioxidants in sudden hearing loss prevention was explained by reducing lipidic–peroxidase synthesis [7,9].

In addition to all these therapies, another effective treatment for sudden hypoacusis is hyperbaric oxygen therapy. It was first introduced by the Marseille School of otology [13]. A recent study showed that an exposure to 100% oxygen at a pressure of 250kPa for 60 minutes improves the results of conventional treatment (Narozny, 2004)[14]. The best results are achieved if the treatment is started as soon as possible. 

## Conclusions

- in our study most of the causes of the sudden hearing loss were of vascular origin;
- the presence of vertigo is a negative prognostic factor;
- the sooner the treatment with vasodilators and corticosteroids begins, the higher are the recovery rates;
- almost 70% of our cases had a good evolution under treatment;
- performing tests for vestibulocochlear system, auditory evoked potentials and MRI (if the above tests are pathologic) to exclude other pathologies;
- the patient reevaluation is necessary after 6 months
